# Man the Winter Carrier of Malaria Organisms

**Published:** 1917-04

**Authors:** 


**Man the Winter Carrier of Malaria Organisms.** M. Bruin Mitzmain, U. S. P. H. S., Bull. No. 84, examined 1183 persons on malarial plantations. 492 infections were identified microscopically. 317 were of the subtertian type, 8 mixed, 1 quartan and the remainder simple tertian. 24.8% of the human carriers harbored gametocytes. 1180 specimens of anopheles were examined during the winter with negative results but 3 anopheles quadrimaculati were found infected in the homes of the gametocyte carriers. The general conclusion is that man and not the anopheles is the winter carrier—and hence, more broadly, the specific carrier of malaria. This fits in with the personal statement of our Associate Editor, Dr. Pel, who told us some years ago that the freedom of Holland from malaria was due to the prompt and general use of quinine to the human hosts.

